# Phaeochromocytoma presenting with polyuria: an uncommon presentation of a rare tumour

**DOI:** 10.1530/EDM-14-0060

**Published:** 2014-10-01

**Authors:** N Atapattu, K A C P Imalke, M Madarasinghe, A Lamahewage, K S H de Silva

**Affiliations:** 1 Lady Ridgeway Hospital, Colombo, Sri Lanka; 2 General Hospital, Matara, Sri Lanka; 3 Department of Paediatrics, Faculty of Medicine, University of Colombo, Colombo, Sri Lanka

## Abstract

Children rarely present with phaeochromocytoma. Their presentation differs from that of adults. The classic triad of sweating, headache and palpitation may not always present in children with phaeochromocytoma. In this study, we present a 6-year-old girl who came to us with polyuria and polydipsia for evaluation of suspected diabetes insipidus. She gave a clear history of increased sweating in the recent past. On clinical examination, she was noted to have high blood pressure. Subsequent investigations revealed a phaeochromocytoma. Her polyuria and hypertension resolved immediately after the surgery. We did not have the facilities to arrange for genetic tests; however, the patient and the family members are under follow-up for other associated conditions.

**Learning points:**

- Polyuria and polydipsia are rare symptoms of phaeochromocytoma.
- Complete physical examination prevented unnecessary investigations for polyuria and led to a correct diagnosis.
- Classic features are not always necessary for diagnostic evaluation of rare diseases.

## Background

Phaeochromocytoma is rare in children (1). The presentation varies from vague symptoms to hypertensive emergencies. The common presenting features are headache, palpitation, diaphoresis, syncope, tremors and weight loss. Anxiety and depression are also described in the literature (2), whereas polyuria and polydipsia are reported to be rare (3). Hypertension in children is generally sustained unlike in adults (4). There is a male predominance of phaeochromocytoma in children. We present a child with phaeochromocytoma who had rare clinical manifestations such as polyuria and polydipsia.

## Case presentation

A 6-year-old girl was referred with a history of polyuria, polydipsia and secondary nocturnal enuresis of 3-month duration. She was noted to be sweaty and her parents have thought she was polydipsic due to excessive sweating. She had been drinking ∼2–3 l of water daily. Urine output corresponded to the oral intake and was documented to be 7 ml/kg per h. She did not have a headache, flushing or recent-onset weight loss, constipation, loss of appetite or visual disturbances.

She was born to non-consanguineous parents with a birth weight of 2.9 kg. She did not have any prenatal or postnatal complications and was apparently healthy till the onset of these symptoms. The family history was unremarkable. On examination, her weight was 17.5 kg (9th centile) and height was 112 cm (25th centile). Apart from hypertension, there was no clinical evidence of coarctation, neurofibromatosis (NF), organomegaly or renal bruit. Blood pressure on several occasions was above the 95th percentile for her age and height (95th percentile – 112/95 mmHg). When her oral intake was restricted to the maintenance requirement, the polyuria resolved. Water restriction was done overnight with careful monitoring of intake and output. Before restricting water intake, the sodium level was checked in the evening and it was 138 mmol/l. At this point, we started restricting fluid overnight. The following morning, the serum sodium level was found to be 140.

## Investigation

**Figure 1:**
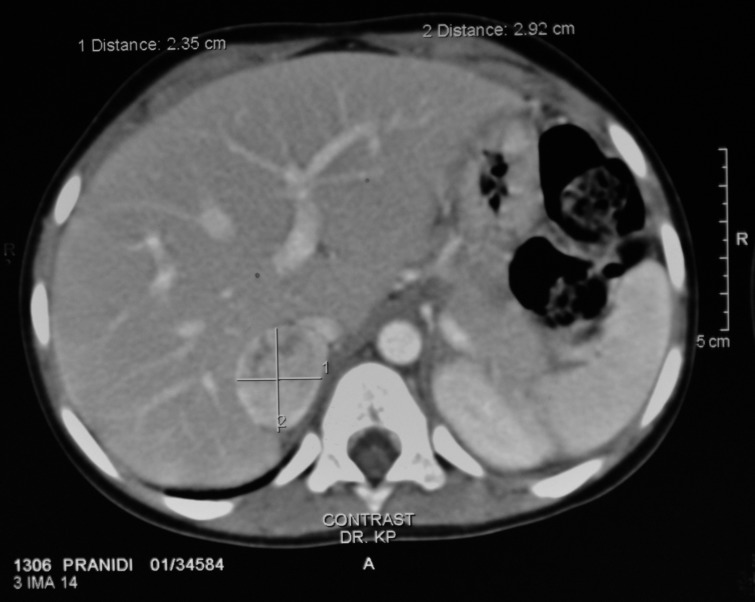
CT abdomen revealing 3.2×2.3×2.9 cm tumour superior to the upper pole of the right kidney.

The blood investigations at the presentation are given in Table 1. Ultrasound (US) scan of the abdomen with Doppler studies did not reveal any evidence of renal artery stenosis, adrenal hyperplasia or adrenal masses. Echocardiogram revealed concentric left ventricular hypertrophy supporting the presence of undetected hypertension. Ophthalmic assessment was normal. The 24 h urinary vanillylmandelic acid (VMA) level was 18.8 mg/24 h (normal range 1–11). We did not analyse urinary or blood metanephrine levels due to non-availability of the tests in the state sector laboratories. Computed tomography (CT) revealed a well-defined lesion of 3.2×2.3×2.9 cm superior to the upper pole of the right kidney (Fig. 1).

**Table 1:** Initial biochemical investigations

	
Serum osmolality	292 mOsmol/l
Urine osmolality	672 mOsmol/l
Na^+^	141 mmol/l
K^+^	4.5 mmol/l
Calcium	2.68 mmol/l
Phosphate	1.58 mmol/l
Alkaline phosphatase	432 IU/l

**Figure 2:**
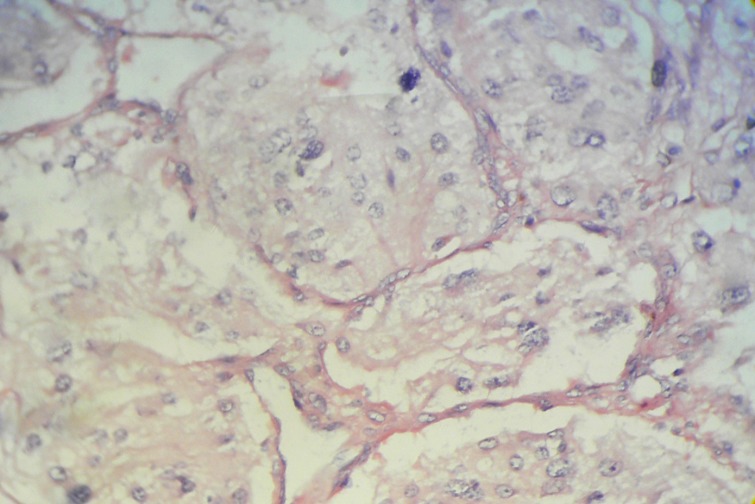
Histology of the tumour showing cells with vascular pleomorphic nuclei and abundant cytoplasm in a nested arrangement.

Histology revealed an adrenal medullary tumour comprising cells with vascular pleomorphic nuclei and abundant cytoplasm in a nested arrangement. Mitotic figures were scanty. Adrenal cortical tissues of normal morphology were observed at the periphery of the tumour (Fig. 2).

## Treatment

She was initially started on prazosin to control the blood pressure. Prazosin has been used effectively according to the literature (5).

Phenoxybenzamine was added when the VMA levels were available, only 3 days before the surgery. Blood pressure was under control within 7 days of starting prazosin. She was prepared for surgery with i.v. fluids to minimise postoperative hypotension. Phenoxybenzamine was stopped 24 h before surgery and prazosin was stopped on the day of surgery. During surgery, her systolic blood pressure was maintained in the range of 110–120 mmHg. Postoperatively, her blood pressure was normal. She did not develop postoperative hypoglycaemia.

## Outcome and follow-up

Blood pressure and polyuria settled following surgery. Both parents and patient underwent calcium measurement and US scan of the thyroid to look for any associated features of multiple endocrine neoplasia (MEN) syndrome. Genetic tests would have helped to do more focused investigations. In the absence of genetic studies, the patient and family members are followed up for headache, hypertension and hypercalcaemia.

## Discussion

Phaeochromocytomas are tumours arising from catecholamine-producing chromaffin cells in the adrenal medulla resulting in excessive production of catecholamines. Symptoms depend on the amount of catecholamines released and the sensitivity of the individual patient (3) and are often related to hypertension such as sweating, visual disturbances, nausea or vomiting in children (3) as opposed to adults. Adrenaline and noradrenaline are the main catecholamines produced by a phaeochromocytoma. Noradrenaline has α excitatory effects but adrenaline has both α and β excitatory effects. Noradrenaline can cross the blood–brain barrier slowly and inhibit antidiuretic hormone (ADH) release (6), which was the probable reason for polyuria and polydipsia in this child. ADH release is mediated by both osmolar and nonosmolar stimuli (7). Experiments have revealed that noradrenaline-induced inhibition of ADH release worked through a baroreceptor-mediated mechanism (8). This baroreceptor-mediated inhibition of ADH release can be blocked with an α adrenergic antagonist, indicating that inhibition of ADH by noradrenaline acts through α adrenergic receptors (9). Elevated blood pressure also inhibits ADH through peripheral baroreceptors by increased sympathetic stimulation (7). The experiments have revealed that denervation of baroreceptors abolished the diuretic effect of i.v. noradrenaline.

There are few case reports in the literature reporting polyuria as a presenting symptom in phaeochromocytoma. Hypertension was detected just before vasopressin challenge in one of the case reports in India (10). Not only children but also adults have presented with polyuria associated with phaeochromocytoma (11). In this case study, there was cerebral venous sinus thrombosis considered to result from paraneoplastic procoagulant substances secreted by the tumour cells.

Germline mutations are observed in 70% of children with a phaeochromocytoma who present before 10 years of age (1). Von Hippel–Lindau (*VHL*) gene is the most commonly mutated gene followed by mutations in subunits B and D of succinate dehydrogenase (*SDHB* and *SDHD*) gene, *RET* proto-oncogene predisposing to MEN2 and the NF type 1 (*NF1*) gene (1). All four types of germline mutations are inherited in an autosomal dominant manner. In the absence of facilities to screen the index case for the genetic mutation, family members were screened for possible associated conditions. Both parents were clinically normal and had normal blood pressure and calcium levels.

Facilities were only available to measure the urinary VMA level. However, the British Society of Paediatric Endocrinology and Diabetes recommends demonstrating elevated urinary metanephrines and catecholamines on two occasions (12).

In the absence of facilities in our country to perform functional imaging with ^123^I labelled metaiodobenzylguanidine (MIBG) scintigraphy, we performed a CT scan to localise the lesion. Owing to radiation risk we only imaged the abdomen and were able to localise the growth. Recurrent symptoms with inability to localise the tumour with CT or magnetic resonance imaging, is a definite indication for an MIBG scan.

Malignancy cannot be predicted on radiological studies and it is not recommended to perform biopsy on the lesions before surgery (5). This child had no evidence of malignancy on histology. Malignancy is rare in phaeochromocytoma according to the British tumour registry and is 0.02 per million per year (5). The child and her parents will be followed up in the clinic in order to detect any recurrence of phaeochromocytoma in the patient or features of a genetic disorder that is associated with phaeochromocytoma in this family.

This case highlights the importance of proper clinical examination of a patient irrespective of the presenting complaint. Her polyuria settled with water restriction overnight while she was in the ward. We would have easily missed the high blood pressure and investigated for diabetes insipidus with a water deprivation test or could have labelled the child as having psychogenic polydipsia.

## Patient consent

The consent for publication of this study was obtained from the mother of the child.

## Author contribution statement

Dr N Atapattu contributed to diagnosis and manuscript writing. Dr K A C P Imalke significantly contributed to managing the patient. Dr M Madarasinghe was the referring physician. Dr A Lamahewage was the surgeon. Dr K S H de Silva was the Unit head and contributed to manuscript editing.
